# Elucidating the Role of Neurotensin in the Pathophysiology and Management of Major Mental Disorders

**DOI:** 10.3390/bs4020125

**Published:** 2014-06-13

**Authors:** Mona M Boules, Paul Fredrickson, Amber M Muehlmann, Elliott Richelson

**Affiliations:** 1 Neuropsychopharmacology Laboratory, Mayo Foundation for Medical Education and Research, Mayo Clinic, Jacksonville FL 32224, USA; E-Mails: fredrickson.paul@mayo.edu (P.F.); richel@mayo.edu (E.R.); 2 Department of Psychiatry, University of Florida, Gainesville FL 32611, USA; E-Mail: muehlman@ufl.edu

**Keywords:** neurotensin, schizophrenia, antipsychotic drugs, addiction, autism spectrum disorder, animal models

## Abstract

Neurotensin (NT) is a neuropeptide that is closely associated with, and is thought to modulate, dopaminergic and other neurotransmitter systems involved in the pathophysiology of various mental disorders. This review outlines data implicating NT in the pathophysiology and management of major mental disorders such as schizophrenia, drug addiction, and autism. The data suggest that NT receptor analogs have the potential to be used as novel therapeutic agents acting through modulation of neurotransmitter systems dys-regulated in these disorders.

## 1. Introduction

Neurotensin (NT) is a neuropeptide that was originally isolated from bovine hypothalami [1]. In addition to its presence in the central nervous system (CNS), it is also found in the gastrointestinal tract, and the cardiovascular system. Both the central and peripheral effects of NT are mediated through the activation of NT receptors. There are three well-characterized NT receptors, NTS1, NTS2 and NTS3 [2,3]. NTS1 and NTS2 are G-protein-coupled receptors with seven-transmembrane domains and are distinguished based on their sensitivity to the histamine receptor antagonist, levocabastine [4,5,6,7], with NTS1 being insensitive to this drug. NTS2 has lower affinity to NT and is sensitive to levocabastine.

Functionally, NTS1 is coupled to the phospholipase C and the inositol phosphate (IP) signaling cascade (refer to [2,8] for reviews). NTS1 signaling can also be mediated through activation of cyclic guanosine monophosphate (cGMP), cyclic adenosine monophosphate (cAMP), arachidonic acid production, and mitogen-activated protein (MAP) kinase phosphorylation [9,10,11,12,13,14,15,16]. The signaling properties of NTS2 are controversial depending upon the species from which the NTS2 was isolated and the cell system used to evaluate signaling [17,18]. NTS3/sortilin is a single transmembrane receptor domain [3,19] that modulates NT intracellular sorting and signaling processes [19] and has been associated with IP and MAP pathways in glial cells [20].

NT’s role has been explored in several physiological and pathological conditions including pain, central control of blood pressure, eating disorders, cancer, inflammation, and CNS disorders [21,22], This review will address the potential role of NT in the management of major mental disorders with special focus on schizophrenia, substance abuse, and autism.

## 2. Neurotensin and Schizophrenia

### 2.1. Overview

Schizophrenia is a severe psychiatric disorder that afflicts approximately 1% of the population worldwide. It begins in late adolescence or early adulthood and usually continues throughout life. Schizophrenia has varied symptoms including auditory hallucinations and delusions (positive symptoms), inability to care for self, apathy and flattening affect, loss of sense of pleasure, and social withdrawal (negative symptoms). Schizophrenic patients also suffer from cognitive impairment including disturbances in attention, disorganization of thought and speech [23], and depression and social isolation [24]. Because of the wide range of symptoms, patients with schizophrenia have difficulty holding a job or caring for themselves, placing a significant burden on their families and society. Additionally, they experience a 20% shorter life span than the general population and are at an increased risk for committing suicide [25].

The pathophysiology of schizophrenia is not quite understood. Several hypotheses have been published, the most supported of which is the dopamine hypothesis [26,27]. While originally hyperdopaminergia was emphasized as the cause of schizophrenia [28] an imbalance of dopamine between different brain regions has been since accepted as causative factor in the pathophysiology of schizophrenia [29]. Hyperactivity of the mesolimbic dopamine system has been related to positive symptoms while hypo-activity of the mesocortical dopamine system has been proposed to cause the negative symptoms and cognitive impairment [29,30,31].

More recently the physiology of this disorder has expanded beyond dopamine dysfunction to include the glutamate, serotonin, and nicotinic/acetylcholine systems. The glutamate receptors including the ionotropic N-methyl-D-aspartate (NMDA) receptors and metabotropic glutamate (mGlu) receptors are also co-localized in many areas of the brain that are highly implicated in schizophrenia, such as hippocampus, striatum, and neocortex [32,33,34]. Therefore, disruptions in the glutamatergic circuitry have been hypothesized to play a role in the pathophysiology of the disease [35,36]. These disruptions are thought to be caused by reduced NMDA function on GABAergic neurons in subcortical regions [37]. The glutamatergic hypothesis has been supported by the following: (1) NMDA receptor antagonists precipitate positive, negative, and cognitive schizophrenia-like symptoms in humans and animals [38,39] and exacerbate symptoms of schizophrenia in patients with the disease [40]; (2) the genetic deletion of mGluR5 in mice results in decreased sensorimotor gating, decreased short-term spatial memory, and decreased sensitivity to locomotor deficits induced by NMDA receptor antagonists [41,42,43]. These behaviors are similar to those induced by glutamate hypo-function and are observed in the schizophrenic phenotype [44]; (3) treating rats with mGlu receptor selective negative allosteric modulators results in social interaction deficits, impaired working memory, reduced instrumental learning, and intensification of the effects of NMDA receptor antagonists [33,45,46,47,48]. Additionally, glutamate has been linked to a myriad of processes surrounding cognition, memory and perception [49]. These studies have led to targeting the major excitatory neurotransmitter in the brain, glutamate, as an emerging novel approach for the treatment of schizophrenia. However, clinical trials of drugs targeting glutamate receptors in schizophrenic patients have yet to provide convincing support for this hypothesis, as discussed further below.

### 2.2. Antipsychotic Drugs (APDs)

Although schizophrenia has been attributed to an imbalance in dopamine, recent reports implicate several interacting systems including the dopaminergic [50], serotonergic, glutamatergic, cholinergic, and GABAergic systems. Nonetheless, most of the currently available APDs mainly target the dopamine D2 receptors [51] or the serotonergic receptors [52,53,54]

The first generation, or “typical”, APDs derive their therapeutic ability mainly through antagonizing the high affinity dopamine D_2_ receptors [55]. Typical APDs are effective at reducing the frequency and severity of psychotic episodes, [56]; however, they fail to eliminate positive symptoms of schizophrenia and in most cases have little effect on the negative and cognitive symptoms, (e.g., [57]) Moreover, typical APDs carry a substantial risk for producing extrapyramidal, cardiovascular, and endocrine side effects [58].

The second generation, or atypical, APDs are characterized by their relative high-affinity for the serotonin 5-HT_2A_ receptor. They are called “atypical”, because they have a low propensity to cause the motor side effects that are typical of the first generation compounds. 5-HT_2A_ antagonism is thought to mitigate the adverse effects of striatal dopamine D_2_ antagonism. It is suggested that a higher ratio of a drug’s affinity for 5-HT_2A_ receptors relative to dopamine D_2_ receptors can predict “atypicality”, indicatinglower risk of extrapyramidal side effects [59] and potential improvement in cognition as compared to typical APDs (refer to [60] for review). However, atypical APDs carry a risk of adverse effects including metabolic syndrome, obesity, sedation, and cardiovascular abnormalities [61]. Agranulocytosis, a potentially fatal condition marked by severely low white blood cell count, has restricted the use of clozapine.

The therapeutic importance of the effects of currently available APDs on dopaminergic neurotransmission is well established. On the other hand, clinical studies with drugs targeting glutamatergic neurotransmission as a mechanism of action of novel APDs are limited and controversial, althoughsuch drugs have shown potential as novel compounds to treat schizophrenia [62]. In one clinical study, patients treated with a metabotropic glutamate receptor agonists showed significant improvement in both positive and negative symptoms [63]. However, these results could not be replicated by Eli Lilly and Company, 2009. Interestingly, chronic administration of clozapine to mGluR5 knockout mice reverses sensorimotor gating deficits, ameliorates hyperactivity but does not improve memory deficits [64]. While a recent study shows that acute, subchronic and chronic treatment with haloperidol and olanzapine do not influence mGluR5 binding density [65], others [66] reported an increase in mGluR5 mRNA expression by haloperidol and sertindole. It is worth noting, though, that mRNA levels do not correlate with protein expression [67] and that APD treatment might affect mGlu receptor function [68]. The effects of APDs on mGlu receptors might also be due to a direct or an indirect interaction with dopamine D2 receptors [69].

Another approach targeting glutamate receptors involves allosterically activating NMDA receptors by targeting their glycine site. This can be achieved with glycine or its analogs or with drugs that inhibit glycine reuptake (glycine transport blockers). Antagonists of glycine transport have also been proposed to have therapeutic potential in treating schizophrenia [70,71]. Glycine transporter 1 (glycine T1) inhibitors have been used adjunctively with APDs to increase their efficacy in treating negative symptoms of schizophrenia [72,73,74]. In addition, clozapine blocks glycine transport and this mechanism may account for its reported advantage over other atypical APDs [75]. Similarly, studies with bioptertin, a non-amino acid derivative of glycine, yielded encouraging findings in decreasing negative symptoms with no major issues of tolerability or toxicity [76]. Thus, the inhibition of the glycineT1 or activation of the glutamate receptors (mGlu5, mGlu2 or mGlu3) might act towards normalizing the disruption of the aberrant signaling in these circuits in schizophrenic patients [77].

While the current APDs have profoundly impacted acute treatment of schizophrenia enormous challenges remain for long-term treatment. The long-term use of typical APDs is associated with the induction of extrapyramidal side effects and the benefits of atypical APDs are tempered by weight gain, metabolic changes, sexual dysfunction, and QTc-prolongation. This leads many patients to non-compliance and discontinuation of treatment [78]. Therefore, there is still a need for therapeutic agents mediating effects through novel targets that provide more effective treatment for schizophrenia with fewer side effects. Evidence suggests that targeting the NT system may provide a novel and promising treatment for schizophrenia.

### 2.3. Neurotensin and Dopaminergic Neurotransmission

NT co-localizes with and modulates the mesolimbic dopaminergic system with more than 80% of dopaminergic neurons expressing NTS1 [79,80,81]. The modulatory effect of NT on dopaminergic neurotransmission depends on the localization of the receptors [82], the threshold of the dopaminergic neurons for NT-induced depolarization [83,84], the amount of NT administered and the site of administration (for a comprehensive review, please refer to [85]).

In the nucleus accumbens (NA), NT receptors are co-localized with presynaptic and postsynaptic dopamine receptors. Pre-synaptically, NT inhibits dopamine D2 autoreceptors resulting in an antagonistic effect on dopamine D2 receptors and an increased firing of dopaminergic neurons via NTS1-mediated increase in intracellular Ca^2+^ [17,86]. On the other hand, post-synaptically NT causes a decrease in dopamine signal transduction in the same area [87]. However, in the dorsal striatum, NT receptors are located almost exclusively on presynaptic dopaminergic terminals causing dopamine D2 autoreceptor inhibition and increasing dopamine signaling [88].

NT also regulates the function of dopamine receptors. NT decreases the binding affinity of dopamine D2 receptors for its agonists [89,90]; up-regulates tyrosine hydroxylase gene expression [91,92]; and alters receptor-dependent second messenger systems, such as phosphorylation and de-phosphorylation equilibrium ([89] for review). At a plasma membrane level, NT also allosterically affects the function capacity of dopamine D2 receptor heterodimers [93].

### 2.4. Neurotensin and Glutamatergic Neurotransmission

The antipsychotic-like effect of NT in animal models has been attributed partially to the increase in the ventral striato-pallidal γ-aminobutyric acid (GABA) transmission. In turn, this causes a restoration of the glutamate levels in the medio-dorsal thalamic nucleus to the prefrontal cortex pathway, which seems to be reduced in patients with schizophrenia [94,95,96].

Intra-cortical perfusion with NT changes extracellular glutamate levels in a bell-shaped and concentration dependent manner indicating that NT plays a relevant role in the regulation of cortical glutamate neurotransmission [97]. Similar *in vivo* and *in vitro* studies indicate the enhancing effects of NT on endogenous glutamate [98,99,100,101,102]. The perfusion of NT in the NA is thought to activate NTS1 receptors located on accumbal glutamate terminals, thereby inducing an enhancement of glutamate outflow that could be associated with a concomitant and significant reduction in accumbal dopamine release [103].

### 2.5. Neurotensin and APDs

APDs affect NT circuits in brain regions that are involved in the pathophysiology of schizophrenia. Acute and chronic administration of APDs increases NT mRNA expression, NT peptide concentration, and NT release in the NA and caudate nucleus [104,105,106,107,108] (Table 1).

**Table 1:** Effects of APDs on the neurotensinergic system.

Effect	Typical APD	Atypical APD
mRNA expression	↑ NT mRNA in DL striatum & NA [104,106,109]	NT mRNA in NA [106,109]
	↑ NT mRNA in neostriatum [107]	
	↑NT mRNA in SN/VTA [110]	No change in SN/VTA [110]
NT receptor binding	↑ NTR binding in SN [104]	↓ NTR binding in SN & NA [104]
	↑ receptor density [111]	↓ receptor density [111]
NT release	↑ NT release in NA & striatum [112]	↑ NT release in NA [112]
NT tissue concentration	↑ NT levels in NA & caudate [104,105,113,114,115,116,117]	↑ NT levels in caudate [114]
	↑ NT-IR in NA [118,119]	
	↑ NT-IR in striatum [120]	↑ NT-IR in Vstriatum & mPFC [120]

DL Striatum = dorso-lateral striatum; NA = nucleus accumbens; NTR = neurotensin receptor; SN = substantia nigra; VTA = ventral tegmental area; NT-IR = neurotensin immune-reactivity; V striatum = ventral striatum; mPFC = medial prefrontal cortex; ↑ = increase; ↓ = decrease.

In addition to the biochemical interaction between NT and APDs, central administration of NT mimics behaviors observed after peripheral administration of APDs. Both induce hypothermia, analgesia, and spontaneous hypo-activity [121,122,123]. NT also reproduces the behavioral response of APD in animals used to evaluate these drugs’ actions [124], such as blockade of apomorphine-induced climbing and physostigmine-induced yawning [125,126], increased vacuous chewing movements [127], decreased avoidance behavior [128], and reversal of drug-induced disruption of prepulse inhibition (PPI) of the acoustic startle response [129]. The PPI of the acoustic startle reflex is defined as a decrease in the startle reflex induced by a strong acoustic stimulus when preceded by a weak prepulse. It measures pre-attentive sensorimotor gating (for review see [130]). These results strengthen the hypothesis that NT acts as an endogenous APD [131].

### 2.6. Neurotensin Analogs in Animal Models of Psychosis

Because of the close association of NT with dopaminergic neurons, NT’s neuro-modulatory effects on the dopaminergic system, and data to suggest that APDs act through their effects on endogenous NT [132], this peptide has been hypothesized to be of therapeutic value in the treatment of schizophrenia. Since NT is rapidly degraded by peptidases when administered peripherally many laboratories, including ours, have been developing NT analogs that are resistant to peptidase degradation. These analogs have been used to elucidate the therapeutic efficacy of NT in animal models of schizophrenia. Most of these peptides are NT (8–13) analogs and are 6-amino acids in length. NT and its analogs show efficacy in several animal models of psychosis (Table 2).

**Table 2:** Animal behavioral studies implicating NT in the treatment of schizophrenia.

Study	Reference
Blockade of apomorphine-induced climbing	[125,126,133]
Increase in vacuous chewing movement	[127]
Reversal of drug-induced disruption of PPI	[129,134,135,136,137,138,139,140,141,142]
Decrease conditioned avoidance behavior	[143]
Enhance latent inhibition	[144,145]
Attenuate amphetamine-induced activity	[146]

PPI = prepulse inhibition.

NT analogs inhibit stereotyped apomorphine-induced climbing without affecting sniffing and licking [133,147,148]. NT analogs block amphetamine- and phencyclidine (PCP)-induced hyperactivity [146,148,149], a condition that may reflect both positive and negative symptoms of schizophrenia [40,150]. The NT agonist, NT69L, which is nonselective for NTS1 and NTS2, also attenuates the PCP-induced increase in glutamate in the prefrontal cortex of the rat [149]. Additionally, NT analogs block amphetamine-, dizocilpine-, and DOI-induced disruption in PPI [134,135,136,148,151]. These compounds affect catecholamine, glutamate, and serotonin (5-HT2A) receptors, respectively. These peptides also inhibit conditioned avoidance responding, which is a test with high predictive validity for screening for APDs in rats [152]. The effect of the individual NT analogs in animal models used for screening APDs has been recently reviewed [22].

The use of NT receptor subtype 1 and 2 knockout mice (NTS1^−/−^ and NTS2^−/−^) provided further evidence for the involvement of NT in the pathophysiology of schizophrenia. NTS1^−/−^ mice show higher basal locomotor activity, greater sensitivity to amphetamine-induced hyperactivity, higher mobility in forced swim test and tail suspension test [153,154] and diminished PPI [137]. Conversely, others [138] detected no difference in basal or amphetamine-induced PPI between NTS1^−/−^ and wild type mice and showed an increase in basal PPI in NTS2^−/−^ mice. Biochemically, NTS1^−/−^ mice are hyper-dopaminergic in a way that is similar to the excessive striatal dopamine activity reported in schizophrenia and have lower basal glutamate levels. In addition, mRNA and protein for dopamine D1 and D2 receptors and glutamate NMDA2A receptor subunits are down-regulated in NTS1^−/−^ suggesting possible interactions between NT, dopamine, and glutamate in the prefrontal cortex [155]. The results show promise for the use of NTS^−/−^ mice as a model for schizophrenia.

### 2.7. Neurotensin Levels in Patients with Schizophrenia

Clinical studies in patients with schizophrenia support the hypotheses generated from animal studies with regard to NT. Drug-free schizophrenic patients have reduced cerebrospinal fluid (CSF) NT concentrations [156,157,158,159,160] which appear to be correlated with more severe psychopathology [156,157,159,161]. Effective treatment with APDs normalizes CSF NT concentrations in a subgroup of schizophrenic patients with lower CSF NT [157,159,160,161]. Additionally, this increase in CSF NT concentration is positively correlated with improvement of negative symptoms [159,161]. However, examination of the NT system in human postmortem tissue has produced inconsistent results ([87,162] for review) (Table 3).

**Table 3:** Human studies implicating NT in the pathophysiology of schizophrenia.

Finding	Reference
↓ CSF NT levels in drug free schizophrenic patients	[156,157,158,159,160]
↓ in CSF NT levels correlated with severe psychopathology	[161]
↑ CSF NT levels positively correlated with improving negative symptoms of schizophrenia	[159,161]

↑ = increase; ↓ = decrease.

### 2.8. Potential Side Effects of NT Analogs

NT analogs with higher selectivity to NTS1 cause transient hypothermia and hypotension (refer to [22] for review).

In summary, the data suggest that NT neurotransmission plays a role in the pathophysiology of schizophrenia and the mechanism of action of APDs. Additionally, animal studies provide rationale for pursuing the development of NT analogs as novel therapeutic approaches for the treatment of schizophrenia. Such use of NT analogs may also have other benefits, which include: (1) attenuating smoking (please see section on NT and substance abuse) that is prevalent in schizophrenic patients [163]; (2) being weight neutral, unlike most atypical APDs, which cause weight gain. Indeed weight loss may occur [164,165,166]; and (3) having no motor extrapyramidal side effects as is seen with typical APDs [133] as NT does not only affect dopamine but also modulates several neurotransmitter systems similar to the effects of atypical APDs.

## 3. Neurotensin and Substance Abuse

### 3.1. Overview

The cycle of addiction involving acquisition, maintenance, withdrawal, and relapse is associated with persistent changes in the brain, particularly the mesocorticolimbic dopamine system (the reward pathway). This circuit projects from the ventral tegmental area (VTA) to the NA, olfactory tubercle, frontal cortex, and amygdala [167]. NT, because it is co-localized with dopamine neurons and modulates dopamine transmission, is a candidate for a regulatory role in reward and addiction [87]. This review will focus on NT’s involvement in addiction to psychostimulant drugs, nicotine and alcohol [168,169,170].

### 3.2. Neurotensin and Psychostimulants

Psychostimulant drugs include cocaine, amphetamine, methamphetamine, methylphenidate, and nicotine. Behavioral sensitization, an animal model for addiction to this class of drugs [171,172], is a process by which the same dose of drug produces increasing degrees of locomotor effects with repeated administration [173]. Locomotor sensitization depends on activation of the mesolimbic dopamine system with additional long-term influences on glutamate, GABA, k-opioid, and other neurotransmitter systems [174]. Initiation of sensitization is associated with changes in the NA shell, while maintenance of sensitization involves the NA core [175,176]. NT’s effects on reward pathways depend on site of action. For example, when injected into the VTA, NT produces hyperactivity and dopamine release in the NA, similarly to effects of psychostimulants [177]. In contrast, NT applied directly to the NA reduces the response to psychostimulants, similarly to that of brain-penetrating NT analogs given extracranially [178].

### 3.3. Nicotine

The NT analog NT69L blocks initiation and sensitization to nicotine [179,180], consistent with an antagonism of nicotine’s psychostimulant effects. In a study of nicotine self-administration, rats were treated with either NT69L or saline once they achieved a stable level of responding to nicotine. Pretreatment with NT69L attenuated nicotine self-infusion under FR1 (fixed ratio of 1) and FR5 schedules of reinforcement [181]. Taken together, these studies are consistent with a potential role for a NT agonist to treat nicotine dependence.

### 3.4. Cocaine

NT is also a candidate for regulation of cocaine’s rewarding properties. However, NT knockout (KO) mice did not differ from wild type (WT) or heterozygous mice in most measures of locomotor activity and conditioned place preference paradigms [182], suggesting a limited role for NT in cocaine addiction. In contrast, NT mediates the dopamine D1 receptor potentiation at GABA_A_ synapses in the oval bed nucleus of the stria terminalis. These changes were positively correlated with motivation in rats to self-administer cocaine [183]. Studies with NT antagonists have shown mixed results. When tested against cocaine-induced locomotor sensitization, the NT antagonist SR48692 given intraperitoneally decreases locomotor activity, but only if given over a two-week period [184]. In contrast, injections of SR 48692 into the NA shell enhance cocaine self-administration in a reinstatement paradigm [185]. NT (8–13) microinjected into the ventral pallidum (VP) reverses cocaine-induced decrease in GABA release, and attenuates cue-induced reinstatement, but paradoxically potentiates cocaine-primed reinstatement [186]. In summary, concerning NT-cocaine interactions, studies show inconsistent results; limited effects, synergy, or antagonism. Some of the contradictions stem from localized *vs.* whole brain interventions. In addition, SR 48692, a NTS-selective antagonist, has agonist properties at NTS2 [187]. Additional studies with whole brain exposure to NT agonists will be of interest to determine whether NT analogs may play a role in management of cocaine dependence.

### 3.5. Amphetamine

Amphetamine administration results in NT release in several brain regions including VTA, NA, and prefrontal cortex [188,189]. Studies with NT antagonists in addiction models with amphetamine have shown varying results. Injection of the NT antagonist SR 142948A into the VTA prior to amphetamine exposure prevent the development of sensitization. However, systemic administration of SR 142948A had no effect, underscoring the fact that local NT effects may not predict whole brain responses [190]. In another study, animals treated with SR 48692 displayed higher rates of locomotor response on day seven, but not day one, compared to those treated with amphetamine alone [191]. Additionally, when SR 48692 was given intraperitoneally, chronic NT blockade significantly reduced locomotor sensitization to amphetamine in rats, but at doses higher than usually required for blockade of acute NT effects [192]. In mice, the effect of a single dose of SR 48692 was able to block expression of amphetamine sensitization [193]. NT agonists have also been shown to block effects of amphetamine, although many of the studies have employed amphetamine challenge in animal models of schizophrenia, rather than animal models of addiction. However, in the majority of those studies, NT agonists have shown antipsychotic-like activity [22], which argues against NT as a primary mediator of amphetamine’s addicting effects. A NT analog, NT69L, blocks d-amphetamine-induced hyperactivity in rats [146]. Another brain-penetrating NT analog, PD149163, significantly reduced locomotor effects of amphetamine, and the effectiveness of the NT analog was not diminished after nine consecutive daily administrations [194].

### 3.6. Methamphetamine

Methamphetamine presumably exerts many of its highly addictive effects via basal ganglia dopamine systems [195], and also influences NT content in these structures. In one study NT content rose 210% in dorsal striatum and 202% in substantia nigra in a contingent response paradigm; significantly more than the non-contingent response to methamphetamine in yoked control animals [196]. In that same study, animals were pretreated with either the NT agonist PD149163 (given subcutaneously) or the NT antagonist SR 48692. Lever pressing decreased dramatically following agonist administration, but was unchanged by antagonist. Interestingly, in another study, low dose methamphetamine (0.5 mg/kg) almost doubled NT concentrations in medial striatum and NA; however, high dose methamphetamine (15 mg/kg) did not alter extracellular NT in these structures, compared to pretreatment levels [197]. One clinical implication of these findings according to the authors is that loss of inhibitory influence of endogenous NT after high dose methamphetamine exposure may lead to unchecked dopamine excess and contribute to psychotic symptoms seen in abusers. In a study of human postmortem brain tissue in chronic (greater than one year) methamphetamine abusers, NT levels were reduced compared to those for matched controls in caudate and putamen, and unchanged in NA [198]. Results are in contrast to that seen in most animal studies, but may reflect adaptation that occurred with long-term methamphetamine abuse.

### 3.7. Neurotensin and Alcohol

Several studies link NT to alcohol abuse. NTS1^−/−^ mice demonstrate increased alcohol consumption compared to that for wild-type littermates when given free choice between alcohol and water, an effect blocked in wild type but not knockout animals by NT69L [169]. In this study, NTS1^−/−^ mice were relatively insensitive to alcohol-induced ataxia, supporting the theory that NTS1 mediates alcohol intoxication and consumption. Comparable studies in NTS2 knockout mice showed decreased sensitivity to hypnotic effects, along with increased consumption of alcohol, but no differences were observed between knockout and wild type animals on ataxic effects. NT69L reduced alcohol intake in both NTS2^−/−^ and wild-type mice [170]. Rats bred to be alcohol-preferring show a more arousing response to alcohol, behaviorally and on electroencephalogram (EEG), compared to non-preferring animals. These findings are similar to responses of human alcohol abusers who also experience less sedation and more arousal from alcohol than do social drinkers. Lower concentrations of NT were found in frontal cortex of alcohol-preferring rats, and differences in EEG frequencies between the groups were attenuated by administration of NT [199]. NT69L, which acts at both the high affinity NTS1 and lower affinity NTS2, predominately acts to reduce alcohol consumption through NTS1. NT69L modulates changes in dopamine and glutamate produced by alcohol in the striatum [200].

A growing body of evidence supports a role for NT in addiction, particularly for psychostimulants and alcohol. However, NT’s actions are complex and sometimes seemingly contradictory, depending on site of administration. Both NT agonists and antagonists have shown potential utility in addiction models. However, at least for psychostimulant addiction, effectiveness of NT antagonists has generally required chronic treatment, and chronic blockade of the receptors may lead to receptor up-regulation, increased NT synthesis and release [201]. Further studies with NT agonists given extracranially will help to clarify whether such compounds are useful therapies for substance use disorders. Table 4 summarizes animal behavioral studies implicating NT in the treatment of drug addiction.

**Table 4:** Animal behavioral studies implicating NT in the treatment of drug addiction.

Study	Reference
*Nicotine*	
Blockade of nicotine-induced hyperactivity	[179]
Blockade of initiation and expression of sensitization to nicotine	[180]
Blockade of nicotine self-administration	[181]
Attenuate nicotine self-administration in alcohol-dependent rats	[168]
*Psychostimulants*	
Attenuate amphetamine-induced activity	[146]
Attenuate cocaine-induced hyperactivity	[146]
Decrease lever pressing for methamphetamine	[196]
*Alcohol*	
Decrease alcohol intake	[169,170]

## 4. Neurotensin and Autism

### 4.1. Overview

Autism Spectrum Disorder (ASD) is a neurodevelopmental disorder that is diagnostically classified based on the persistence of both social behavioral deficits and the presence of restricted, repetitive behaviors [202]. These restricted and repetitive behaviors can vary greatly amongst individuals with ASD and can range from simple motor stereotypies and self-injurious behavior to insistence on sameness/routines and restricted, circumscribed interests [203]. These patterns of behavior are extremely inflexible in individuals with ASD and they impair daily functioning. Unfortunately, our understanding of the neuropathology that mediates these repetitive behaviors is limited and our strategies for pharmacological treatment are equally inadequate. Given that a vast majority of mental illnesses are best treated with a combination of two or more therapy types, reducing repetitive behavior with pharmacotherapy may improve responses to behavioral and occupational therapies for the other symptoms of ASD.

Phenotypic heterogeneity within the repetitive behavior domain and across the other aberrant behavioral characteristics associated with ASD (e.g., irritability, impulsivity, and aggression) has made the study of the neuroanatomical and neurochemical bases for these behavioral disorders very difficult. Moreover, this phenotypic diversity also significantly complicates clinical trials for novel pharmacological treatment [204]. As such, psychiatrists have been confined to only a few classes of drugs to try to reduce restricted, repetitive behaviors and none of these has proven safe and effective for a large majority of individuals with ASD. APDs are commonly prescribed to individuals with ASD and both risperidone and aripiprazole are FDA-approved for the treatment of irritability. These drugs are also commonly prescribed “off-label” (non-approved by FDA) for the reduction of impulsivity, aggression, and repetitive behaviors. As stated previously, side effects of APDs include weight gain, motor side effects, and sedation. The marginal efficacy for any pharmacological treatments of repetitive behaviors in individuals with ASD leaves much to be desired and suggests that we must improve our understanding of the neuropathology that mediates repetitive behaviors in order to elucidate more targeted compounds for repetitive behavior remediation.

### 4.2. Neurotensin, Repetitive Behavior, and Basal Ganglia Circuitry

As we have discussed, NT is described as an endogenous antipsychotic drug based on its close association with dopaminergic neurotransmission systems and its effects in animal models that predict antipsychotic effects in humans. Specifically, intracerebroventricular administration of both NT and APDs reduce psychostimulant-induced hyperactivity [205] and APDs increase NT concentrations in NA and dorsal striatum [118], effects that might confer the biological basis of the therapeutic response.

The NT system is also closely associated with glutamatergic and GABAergic neurotransmission. These three neurochemical systems—dopamine, glutamate, and GABA—all descend and interact in the basal ganglia nuclei. Cortico-basal ganglia circuitry mediates repetitive behavior, as demonstrated by neuroimaging studies in the diverse patient populations that exhibit repetitive behavior, including ASD [206,207,208,209,210,211,212,213]. Administration of NT causes increased release of glutamate and GABA from neurons in the cortex, striatum, and globus pallidus [97,214]. It also causes depolarization of indirect basal ganglia pathway neurons [215].

### 4.3. Animal Models of Repetitive Behavior

Repetitive behavior is exhibited in a wide variety of species and across a wide range of environments (though impoverishment and restriction are common traits across these environments). These abnormal behaviors can also be the consequence of genetic and pharmacological manipulations. Knockout mouse models that alter excitatory synapse development and structure commonly exhibit repetitive behaviors [216,217,218,219], as do rodents being administered dopamine and glutamate agonists. These findings further support the role of cortico-basal ganglia circuitry dys-regulation in repetitive behavior. In addition, environmental restriction models of repetitive behavior with the use of both inbred and outbred strains of mice have revealed significant indirect basal ganglia pathway dysfunction [220,221,222]. This dysfunction can be reversed by both pharmacological manipulations and environmental enrichment that change indirect basal ganglia pathway function [223,224,225]. Based on the interactions between NT signaling and indirect basal ganglia pathway function, we hypothesize that NT targeted drugs may offer significant pharmacological efficacy for reduction of repetitive behaviors in both animal models and individuals with ASD.

### 4.4. Neurotensin and ASD

NT has not received much attention as a possible factor in the pathophysiology or in the treatment of ASD [226]. One small study found significantly higher levels of serum NT in children with ASD relative to control children [227], though a correlation with repetitive behaviors or any other ASD-related behaviors was not achieved. In fact, elevated NT levels in the periphery may play a role in the dys-regulated peripheral systems that are associated with ASD, including immune reactivity, gastrointestinal problems, feeding difficulties, and sensory processing. NT stimulates the release of cellular mitochondrial DNA, which is elevated in serum of children with ASD [228]. Extracellular mitochondrial DNA, induced by NT release, causes elevation of the same cytokine precursors and cytokines (e.g., IL-6, NF-kB; [229]) that are overly expressed in the brain of individuals with ASD [230]. In addition, gastrointestinal symptoms correlate with ASD severity [231] and NT serves various functions in the gastrointestinal tract [232]. NT is also an anorexigenic neuropeptide [233] that may contribute to the feeding problems that are commonly exhibited by children with ASD [234]. Lastly, NT transmission mediates opioid-independent analgesia, as well as stimulates the stress-related hypothalamic-pituitary-adrenal (HPA) axis, two systems that are dys-regulated in some children with ASD [235].

Taken together several lines of evidence lead us to hypothesize that NT systems may be dys-regulated in ASD and may offer a target for clinically effective reduction in ASD symptoms. As is the case with drug addiction, the actions of NT are complex and the direction of change needed in NT function may be different in the CNS *versus* the periphery. Furthermore, the regulation of membrane receptors following NT-based treatments is multifaceted [2] and differs across acute and chronic administration protocols and NT receptor subtypes [236,237]. As such, it will be important to decipher the role of an agonist or antagonist treatment in cell body or terminal regions and which receptor subtypes will be most effective as targets to reduce repetitive behaviors. Targeting NT heteromeric receptor complexes (e.g., NTS1/NMDA and NTS1/D2) may also prove to be a valid pharmacological approach. Although many questions still remain, pursuing NT-targeted drugs may offer better therapeutic efficacy for treatment of repetitive behaviors over the commonly prescribed APDs and may also be of benefit treating other non-CNS problems commonly reported in individuals with ASD.

## 5. Conclusions

NT is a tridecapeptide that is found in the CNS. It behaves as a neurotransmitter in the brain and acts as a neuromodulator to several neurotransmitter systems including dopaminergic, sertonergic, GABAergic, glutamatergic, and cholinergic systems. Due to its association with such a wide variety of neurotransmitters, NT has been implicated in the pathophysiology of major mental disorders such as schizophrenia, drug abuse, and autism.

The use of NT analogs, that can be injected systemically, can cross the blood-brain barrier, show promising effects in animal models of psychosis without causing signs that are predictive of extrapyramidal side effects. Additionally, NT analogs are effective in blocking hyperactivity caused by acute administration of d-amphetamine, cocaine, nicotine, and alcohol in rats and mice, as well as sensitization to these psychostimulants. NT analogs also attenuate nicotine self-administration and reduce alcohol consumption. Furthermore, recent studies suggest that the NT system may be dys-regulated in ASD and preliminary data show encouraging effects of NT analogs in treating repetitive behaviors characteristic of ASD in mice. NT analogs hold great promise as a therapeutic agent for schizophrenia, psychostimulant abuse, and ASD.
